# PD-1 inhibitor combined with albumin paclitaxel and apatinib as second-line treatment for patients with metastatic gastric cancer: a single-center, single-arm, phase II study

**DOI:** 10.1007/s10637-024-01425-3

**Published:** 2024-02-12

**Authors:** Miaomiao Gou, Yong Zhang, Zhikuan Wang, Niansong Qian, Guanghai Dai

**Affiliations:** 1 Medical Oncology Department, The Fifth Medical Center, Chinese People’s Liberation Army General Hospital, Haidian District, Fuxing Road 28, Beijing, 100853 People’s Republic of China; 2 Medical Oncology Department, The Second Medical Center, Chinese People’s Liberation Army General Hospital, Beijing, People’s Republic of China; 3 Respiratory and Critical Care Medicine Department, The Eighth Medical Center, Chinese People’s Liberation Army General Hospital, Beijing, People’s Republic of China

**Keywords:** Albumin paclitaxel, Endpoint, Metastatic gastric cancer, Second line

## Abstract

**Background:**

Immune checkpoint inhibitors have been approved for first- and third-line treatment of advanced gastric cancer. However, pembrolizumab alone in the second line did not improve overall survival compared to chemotherapy in the KEYNOTE-061 study. In this study, we aimed to explore the efficacy and safety of a three-drug regimen of PD-1 inhibitor combined with albumin paclitaxel and apatinib (a VEGFR inhibitor) for the second-line treatment of patients with metastatic gastric cancer (mGC).

**Methods:**

This was a single-center, single-arm, phase II clinical study. Patients with mGC with stable microsatellite and negative HER-2 expression who failed first-line chemotherapy were enrolled. The enrolled patients were treated with PD-1 inhibitor (selected according to patients’ requirements) in combination with albumin paclitaxel (125 mg/m^2^, intravenously, days 1 and 8, or 250 mg/m^2^, intravenously, day 1) and apatinib (250 or 500 mg, orally, days 1–21) every 3 weeks. The primary endpoint was progression-free survival (PFS), and the secondary endpoints were overall survival (OS), objective response rate (ORR), disease control rate (DCR), duration of response, and adverse events (AEs).

**Results:**

From July 11, 2019, to October 13, 2022, a total of 43 patients were enrolled, of whom 10 were PD-L1 negative, 11 were PD-L1 positive, and 22 had unknown PD-L1 expression. As of the data cutoff on April 1st, 2023, nine patients had partial response, 29 had stable disease, and five experienced progressive disease, with the ORR of 20.9% and DCR of 88.3%. The median PFS was 6.2 months (95% CI, 3.9–9.3), and the median OS was 10.1 months (95% CI, 7.5–14.1). All patients suffered from alopecia and neurotoxicity. The other main AEs of grade 1 or 2 were bone marrow suppression (*N* = 21, 48.8%), hand-foot reaction (*N* = 19, 44.2%), hypertension (*N* = 18, 41.9%), hypothyroidism (*N* = 11, 25.6%), gastrointestinal bleeding (*N* = 3, 7.0%), and liver function damage (*N* = 5, 11.6%). Two patients reported grade 3–4 immune-related liver damage.

**Conclusion:**

Second-line PD-1 inhibitor combined with albumin paclitaxel and apatinib showed certain efficacy and safety in patients with mGC.

**Trial registration:**

Clinical trials, NCT04182724. Registered 27 November 2019; retrospectively registered, https://clinicaltrials.gov/study/NCT04182724

## Introduction

According to Global Cancer Statistics 2020, over 1 million new cases of gastric cancer (GC) occurred in 2020, which is the 5th most common malignancy and the 4th leading cause of cancer death [1]. Of these, 60% of new cases occurred in East Asia [2]. About 40–50% of patients present with unresectable disease at diagnosis due to a locally advanced or metastatic condition [3]. Among them, about 40% of patients receiving first-line therapy may be candidates for second-line therapy. Oxaliplatin plus with capecitabine (Xelox) or S1 (SOX) were most commonly used in the first-line and subequal taxane agents in the second settings in China. Recently, the management of metastatic GC (mGC) has improved with the advent of novel drugs and the establishment of a continuum of care for this aggressive disease. Immunotherapy has become the mainstay of treatment for mGC according to data from several clinical trials [4, 5]; however, the standard second-line or subsequent therapy is dependent on prior therapy and patient performance status (PS), with survival being limited and frustrating. The National Comprehensive Cancer Network (NCCN) recommended second-line preferred regimens including ramucirumab and paclitaxel, docetaxel, paclitaxel, irinotecan, fluorouracil, irinotecan, and so on. The randomized phase 3 studies RAINBOW [6], REGARD [7], WJOG 4007 [8], and COUGAR-02 [9] showed that median progression-free survival (PFS) in patients with advanced gastric or gastro-esophageal junction (G/GEJ) adenocarcinoma who progressed on first-line chemotherapy with platinum and fluoropyrimidine was less than 6 months and median overall survival (OS) was less than 9 months, representing an unmet need for clinician and patients. Compared to paclitaxel, pembrolizumab, a programmed cell death 1 (PD-1) inhibitor as a kind of immunotherapy, did not significantly improve OS as second-line therapy for advanced GC with a programmed cell death ligand 1 (PD-L1) combined positive score (CPS) of 1 or higher, although pembrolizumab had a better safety profile [10]. Therefore, more effective treatment regimens are required.

In recent years, combination therapy of antiangiogenic agents with anti-PD-1 antibodies has shown a favorable outcome and exhibited a synergistic effect. The anti-PD-1 antibody nivolumab plus regorafenib (a VEGFR1-3 inhibitor) achieved an ORR of 44% (5/9) in patients with pretreated GC in REGNIVO study [11], which provides a rationale for the application of immunotherapy combined with antiangiogenic agents in gastric or esophagogastric junction cancer (GC/EGJC). Combination therapy with a PD-1 inhibitor and apatinib (a domestic highly selective VEGFR1-3 inhibitor) further showed encouraging clinical activity and tolerable toxicity in the second-line setting [12].

As far, there have been no studies on the addition of antiangiogenic agents and anti-PD-1 antibodies to chemotherapy for mGC. In the ABSOLUTE trial [13], weekly nanoparticle albumin-bound paclitaxel (w-nab-PTX) showed non-inferiority to weekly solvent-based paclitaxel (w-sb-PTX) for OS and PFS. Therefore, based on the results of previously developed innovative strategies, we carried out a prospective phase II clinical study to assess the efficacy and safety of a PD-1 inhibitor combined with apatinib and albumin paclitaxel (a surrogate of paclitaxel) as second-line therapy for patients with mGC.

## Methods

### Study design and participants

This was a single-center, single-arm, phase II trial done at the Chinese PLA General Hospital. Key eligibility criteria for patient enrolment included aged 18 years or above, pathological diagnosis of metastatic G/GEJ adenocarcinoma, had at least one measurable lesion, harboring microsatellite stable (MSS)/proficient mismatch repair (pMMR) and HER-2 negative tumors, Eastern Cooperative Oncology Group (ECOG) performance status of 0 or 1, experienced disease progression after first-line therapy with platinum- or oxaliplatin-based chemotherapy, and adequate bone marrow reserve and liver kidney function. Patients were enrolled irrespective of PD-L1 expression. The major exclusion criteria included patients who had received immunotherapy with PD-1 or PD-L1 inhibitor or other drugs; previous treatment with anti-angiogenic agents; active or a history of chronic or recurrent autoimmune disease; or the presence of a serious comorbidity, such as intestinal palsy, intestinal obstruction, pulmonary fibrosis, uncontrolled diabetes, heart failure, myocardial infarction, unstable angina, renal failure, liver failure, mental health disorders, and cerebrovascular disease. The study was registered at ClinicalTrials.gov (NCT04182724). The study was done in accordance with the Declaration of Helsinki and Good Clinical Practice Guidelines, with the approval of the ethics board of the Chinese PLA General Hospital. All enrolled patients provided written informed consent.

### Procedures

Eligible patients received a patient-selected PD-1 inhibitor, apatinib (initial dose 250 mg or elevated dose 500 mg orally on days 1–21 if 250 mg tolerant on previous cycle), and albumin paclitaxel (125 mg/m^2^ on days 1 and 8 or 250 mg/m^2^ on day 1, intravenously) every 3 weeks until disease progression, intolerable toxicity, or for up to 8 cycles. After 8 cycles of treatment, patients with complete response (CR), partial response (PR), or stable disease (SD) received maintenance treatment with a PD-1 inhibitor and albumin paclitaxel. Dose modification was performed according to the patients’ tolerance. All enrolled patients provided written informed consent.

### Assessment and endpoints

Tumors were evaluated every two cycles by the investigator according to RECIST version 1.1. The primary endpoint was PFS (defined as the time from enrollment to disease progression or death from any cause). The secondary endpoints were OS (defined as the time from enrollment to death or censored at the last date of follow-up), objective response rate (ORR, defined as the proportion of patients with complete response [CR] or PR), disease control rate (DCR, defined as the proportion of patients with CR, PR or SD), duration of response (DOR, defined as the time from CR or PR to disease progression or death from any cause), and adverse events (AEs). AEs were graded with the National Cancer Institute Common Terminology Criteria for Adverse Events version 4.0.

### Statistical analysis

The total sample size required to meet the primary endpoint of progression-free survival (PFS) was 40. This is a single-arm phase II study (one-sample log-rank test), to explore clinical activity in terms of PFS with a HR of 0.65. Using the following parameters, median PFS (control arm) = 5 months, *α* = 0.1 (two-sided), *β* = 0.2 (80% power), HR = 0.65, one-sample log-rank test, 48 month recruitment period, 15 month follow-up period, 40 patients, and 38 events were required. Categorical variables were presented as frequency (percentage), and continuous variables were presented as median (range). PFS and OS were estimated using the Kaplan–Meier method. Statistical analyses were done using SPSS (version 21.0). The censoring rules for primary analyses are as follows: (a) No PD and no death, and new anticancer treatment is not initiated, censored at last disease assessment; (b) No PD and no death, and new anticancer treatment is initiated, censored at last disease assessment before new anticancer treatment; (c) PD or death documented after ≤ 1 missed disease assessment, progressed at date of documented PD or death; (d) PD or death documented after ≥ 2 missed disease assessment, censored at last disease assessment prior to ≥ 2 missed disease assessment.

## Results

### Baseline characteristics

From July 11, 2019, to October 13, 2022, a total of 43 patients were enrolled. Baseline characteristics are listed in Table 1. All the patients harbored MSS/pMMR and HER-2 negative tumors, with a median age of 60 years (range, 35–77). Among these patients, 72.1% of patients were male and 27.9% had an ECOG performance status of 0. PD-L1 expression was positive in 25.6% (11 out of 43) of patients, negative in 23.3%, and unknown PD-L1 expression in 51.1%. In addition, 90.7% of tumors were located in the stomach and a minority (9.3%) were in the EGJ. Seventy-nine percent (34 out of 43) had multiple metastatic lesions (≥ 2), and 44.2% (19 out of 43) had liver metastasis. Of the 43 patients, 44% had a history of surgery. The PD-1 inhibitors that the patients received included sintilimab (*n* = 20), pembrolizumab (*n* = 9), nivolumab (*n* = 9), and camrelizumab (*n* = 5).

**Table 1:** Baseline characteristics of patients

	**Patients (*n* = 43)**
**Age, years**	
Median (range)	60 (35–77)
**Gender, *n* (%)**	
Female	12 (27.9)
Male	31 (72.1)
**ECOG PS, *n* (%)**	
0	12 (27.9)
1	31 (72.1)
**Primary tumor location, *n* (%)**	
Gastric	39 (90.7)
GEJ	4 (9.3)
**PD-1 inhibitor, *n* (%)**	
Sintilimab	20 (46.5)
Pembrolizumab	9 (20.9)
Camrelizumab	5 (11.6)
Nivolumab	9 (20.9)
**Number of organs with metastases, *n* (%)**	
1	9 (20.9)
≥ 2	34 (79.1)
**Liver metastases, *n* (%)**	
Yes	19 (44.2)
No	24 (55.8)
**PD-L1 expression, *n* (%)**	
Positive	11 (25.6)
Negative	10 (23.3)
Unknown	22 (51.1)
**History of gastrectomy, *n* (%)**	
Yes	25 (58.1)
No	18 (41.9)

*ECOG PS* Eastern Cooperative Oncology Group performance status, *GEJ* gastro-esophageal junction, *PD-1* programmed cell death 1, *PD-L1* programmed cell death ligand 1

### Efficacy

**Fig. 1:**
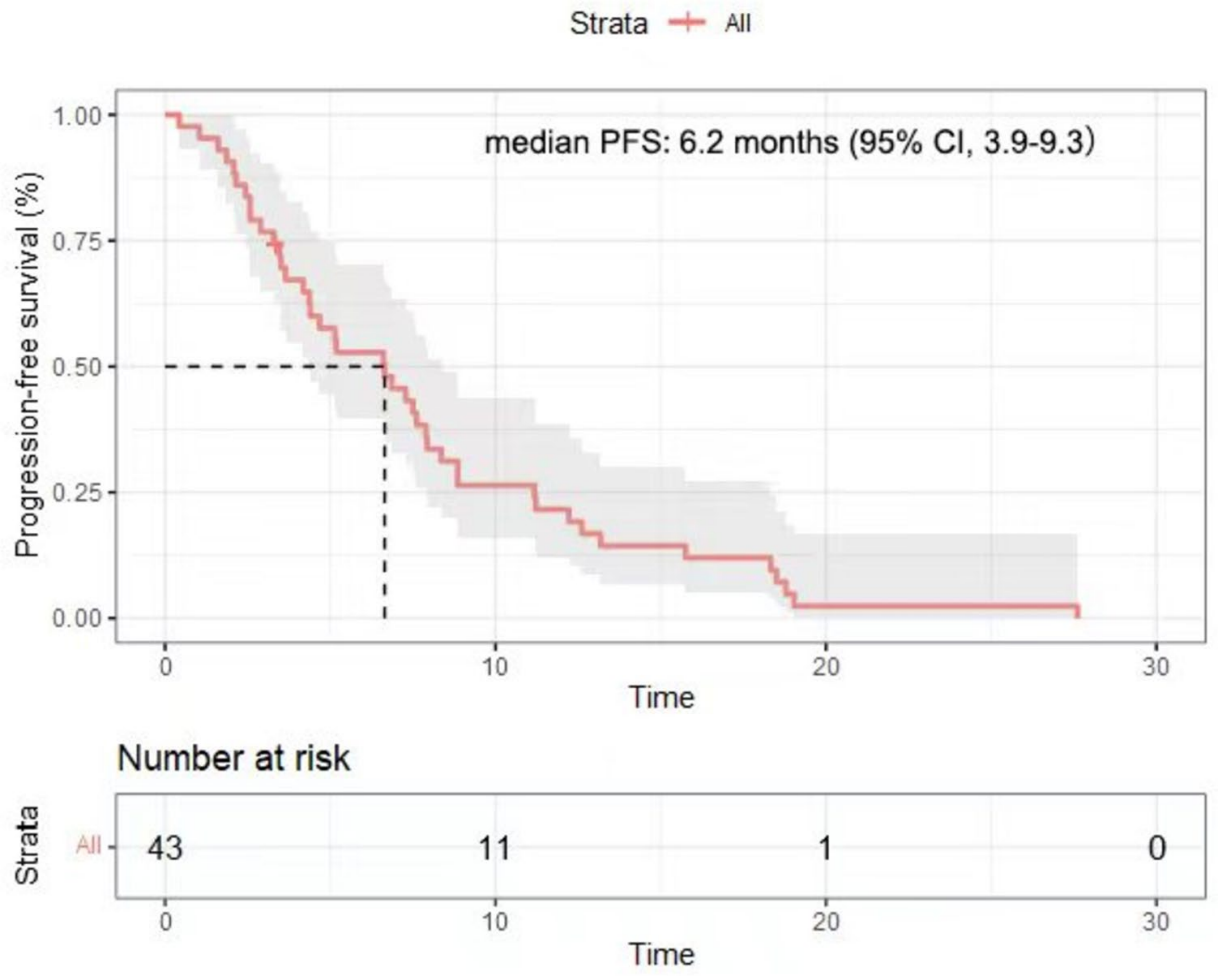
Kaplan–Meier plots of progression-free survival in 43 patients with mGC

**Fig. 2:**
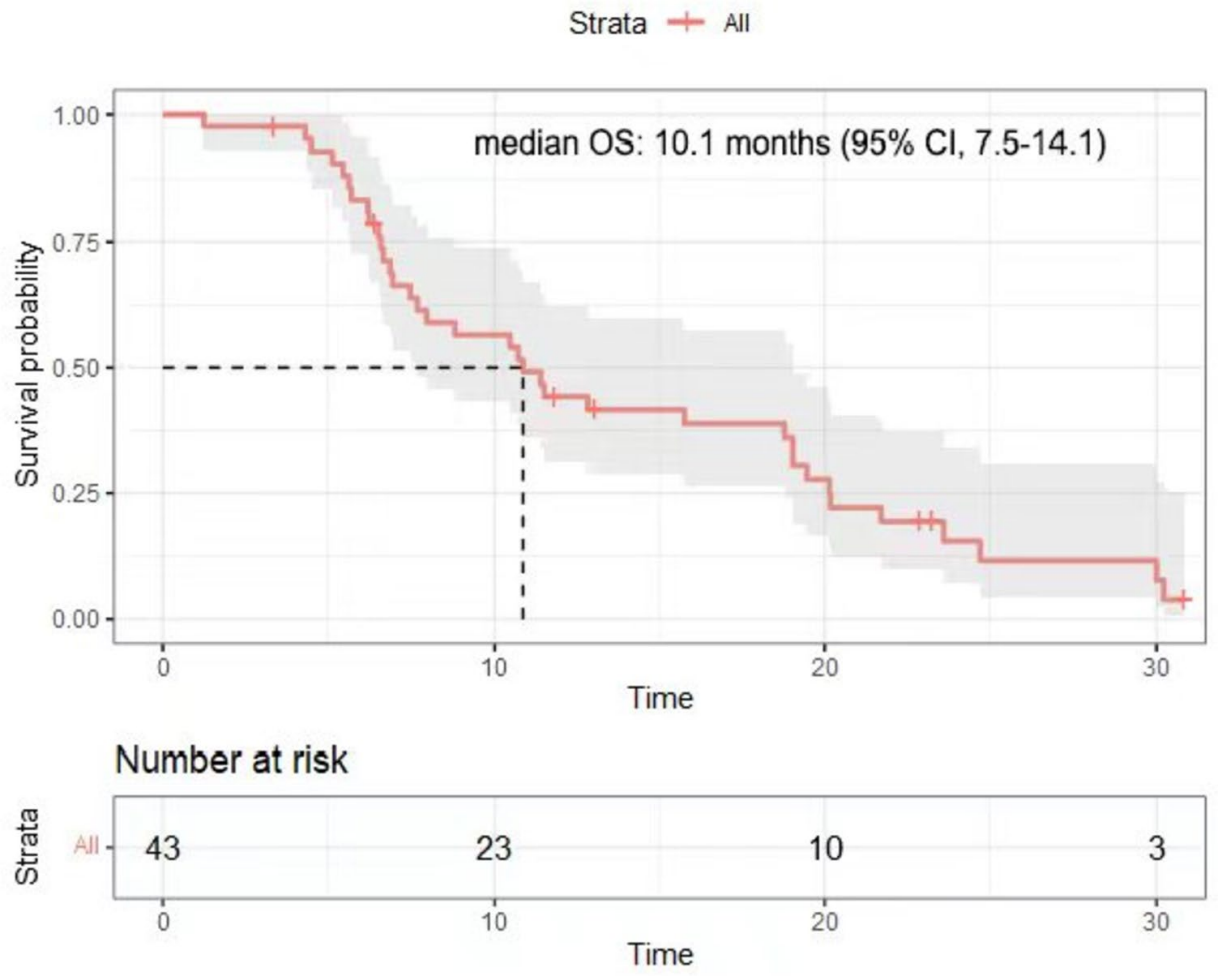
Kaplan–Meier plots of overall survival in 43 patients mGC

The data cutoff for analysis was April 1st, 2023, with a median follow-up of 11.5 months (range, 2.3–30.2). The median number of treatment cycles was 7, and 17 patients received maintenance treatment. At the data cutoff, four patients were still alive and the rest had died. The median PFS was 6.2 months (95% CI, 3.9–9.3, Fig. 1). The median OS was 10.1 months (95% CI, 7.5–14.1, Fig. 2). The 1-year PFS rate was 18.6%, the 1-year survival rate was 46.5%, and the 2-year survival rate was 13.9%. In the subgroup analysis, patients with PD-L1 positive tumors had numerically higher PFS (8.3 months; 95% CI, 1.0–15.6) and OS (19.0 months; 95% CI, 11.0–27.1) than those with PD-L1 negative tumors (PFS, 7.2 months; 95% CI, 6.1–8.4; OS, 10.4 months; 95% CI, 6.3–14.5), although without statistically significant differences (*P* > 0.05).

**Fig. 3:**
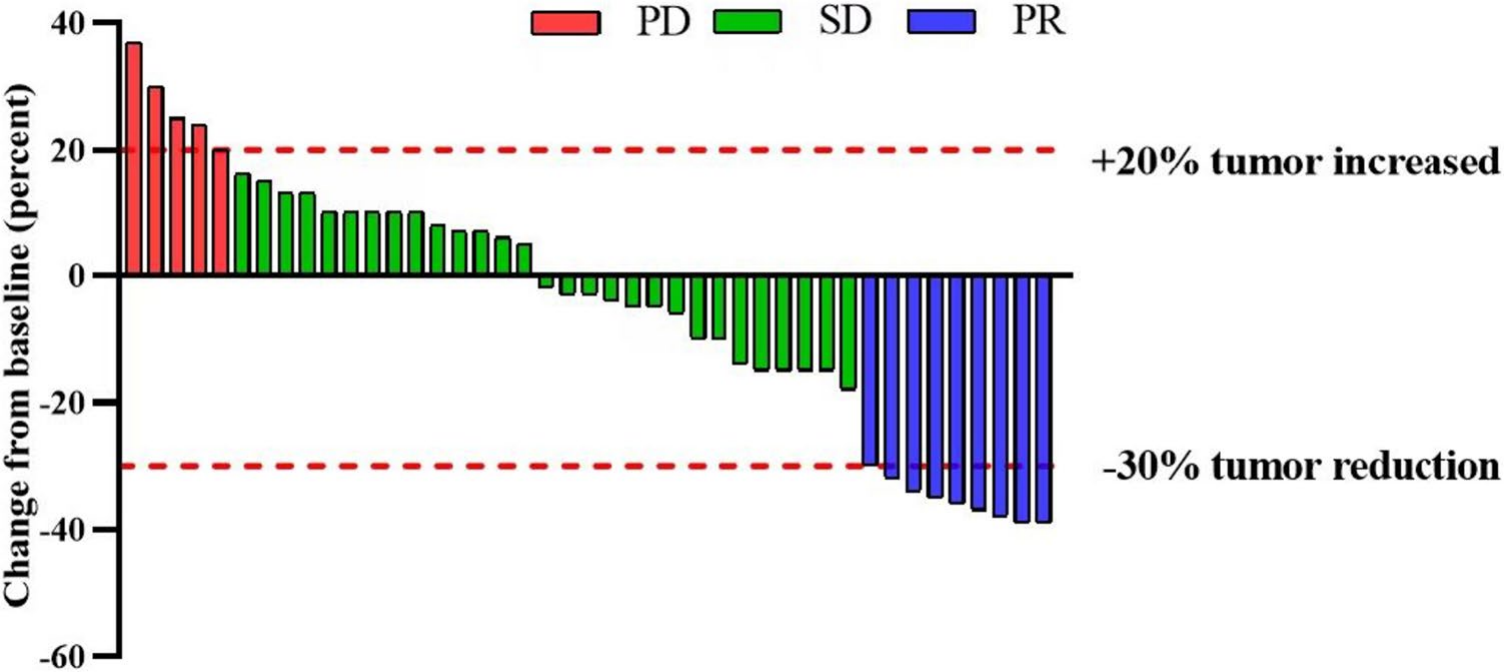
Waterfall plot of best percentage change from baseline in size of target lesion in 43 patients with mGC

**Fig. 4:**
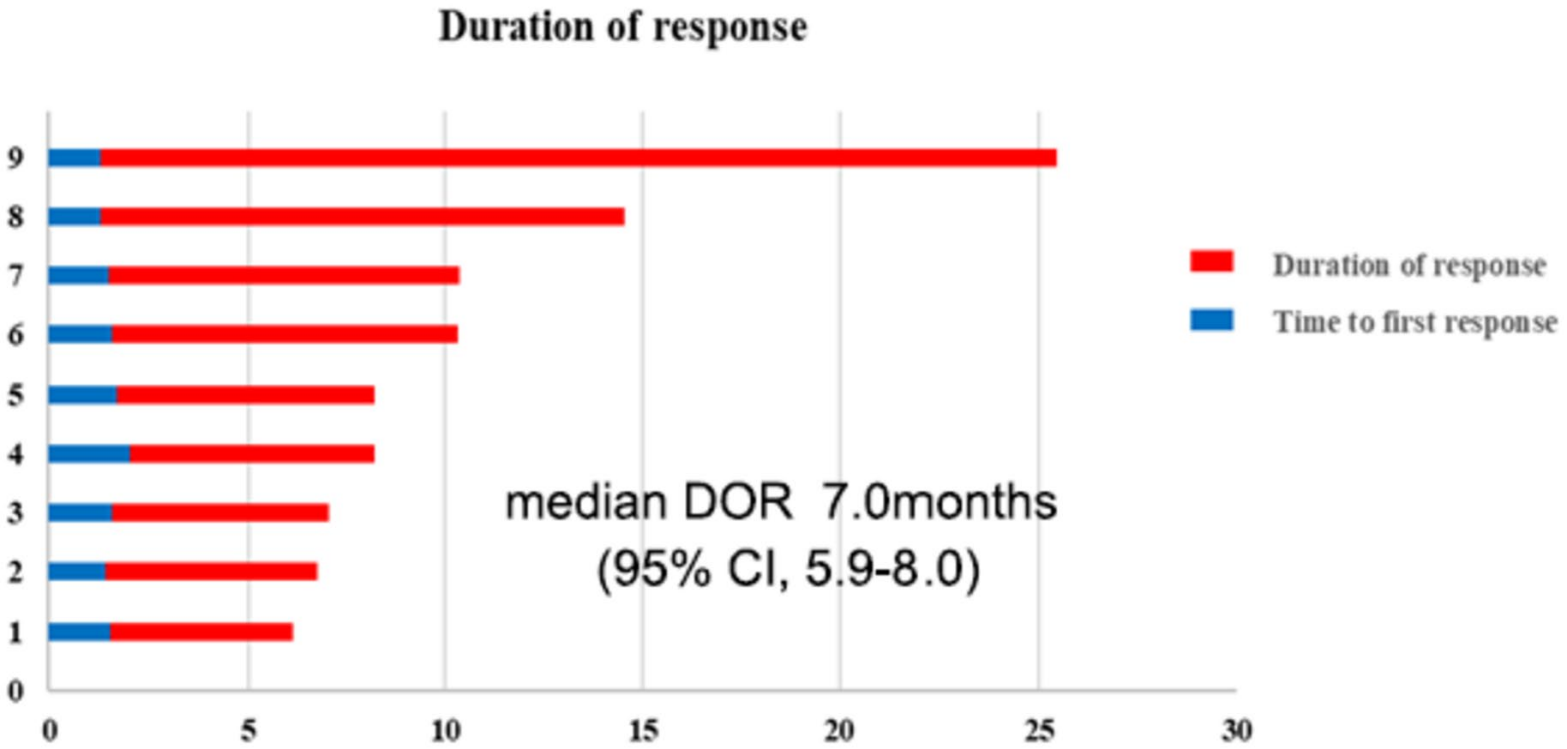
Duration of response for patients with partial response in 43 patients with mGC (blue, time from start to partial response; red, time from response to progression)

An objective response was observed in nine (20.9%) of the 43 patients. Twenty-nine patients achieved SD, while five patients had progressive disease (Table 2). The DCR was 88.3%. Tumor regression is shown in Fig. 3. At the data cutoff, the median DOR was 7.0 months (95% CI, 5.9–8.0), as described in Fig. 4.

**Table 2:** Tumor response

**Tumor response**	**Patients (*n*** **= 43)**
Complete response, *n* (%)	0 (0)
Partial response, *n* (%)	9 (20.9%)
Stable disease, *n* (%)	29 (67.4%)
Progressive disease,* n* (%)	5 (11.6%)
Objective response rate, *n* (%)	9 (20.9%)
Disease control rate, *n* (%)	38 (88.3%)

### Safety

In terms of TRAEs, all patients suffered from at least one AE. As in Table 3, the most common AEs of grade 1 or 2 were alopecia (*n* = 43, 100%), neurotoxicity (*n* = 43, 100%), bone marrow suppression (*n* = 21, 48.8%), hand-foot reaction (*n* = 19, 44.2%), and hypertension (*n* = 18, 41.9%). Grade 3 treatment-related AEs occurred in nine patients, including bone marrow suppression (five [11.6%]), liver function damage (two [4.7%]), and skin rash (two [4.7%]). The liver damage and skin reaction were considered immune-related and mitigated by hormone treatment. Toxicities were generally well tolerated. No previously unreported AEs and severe AEs were observed.

**Table 3:** Treatment-related adverse events (TRAEs)

**Adverse events**	**Grade 1 or 2,** ***n*** **(%)**	**Grade 3 or 4,** ***n*** **(%)**
Alopecia	43 (100.0%)	0 (0%)
Neurotoxicity	43 (100.0%)	0 (0%)
Bone marrow suppression	21 (48.8%)	5 (11.6%)
Hypertension	18 (41.9%)	0 (0%)
Hypothyroidism	11 (25.6%)	0 (0%)
Hand-foot reaction	19 (44.2%)	0 (0%)
Nausea	5 (11.6%)	0 (0%)
Liver function damage	5 (11.6%)	2 (4.7%)
Skin reactions	5 (11.6%)	2 (4.7%)
Proteinuria	5 (11.6%)	0 (0%)
Gastrointestinal bleeding	3 (7.0%)	0 (0%)
Diarrhea	3 (7.0%)	0 (0%)
Pneumonia	2 (4.7%)	0 (0%)

## Discussion

This single arm, single-center, phase II trial evaluated the efficacy and safety of a combination therapy of a PD-1 inhibitor with albumin paclitaxel and apatinib as second-line treatment in patients with mGC, providing an option for patients who were previously treated with first-line platinum- or oxaliplatin-based chemotherapy.

This was the first time that the PD-1 inhibitor and small molecule anti-angiogenic agents were combined with chemotherapy in an attempt to improve the current dilemma of second-line therapy. Nivolumab and sintilimab have been approved as immune checkpoint inhibitors for the first-line treatment of advanced GC/EGJC [4]. But till now, there has been no evidence that PD-1 inhibitor monotherapy is comparable to single-agent chemotherapy [10]. Apatinib was approved as third-line therapy for advanced GC in 2014 [14]. The combination of PD-1 inhibitor and anti-angiogenesis has a coordinated function, which has been demonstrated in several works [12]. However, the efficacy of the addition of chemotherapy to the above combination in the second-line therapy is still questionable.

In our study, it was observed that the median PFS was 6.2 months (95% CI, 3.9–9.3), and OS was 10.1 months (95% CI, 7.5–14.1). Compared with the results of previous studies, a significantly increased survival benefit was shown. These results suggested that the outcomes of the three-drug combination were more favorable than those of double drugs in previous studies. In the RAINBOW study [6], the median PFS with ramucirumab plus paclitaxel was 4.4 months (95% CI, 4.2–5.3) and the median OS was 9.6 months (95% CI, 8.5–10.8). The outcomes were even worse in the REGARD study, with the median OS of 5.2 months and the median PFS of 2.1 months [7]. In the similar population-based study of nivolumab combined with paclitaxel plus ramucirumab as second-line treatment for advanced GC [15], the 6-month PFS rate was 46.5% (80% CI, 36.4%–55.8%), and median survival time was 13.1 months (95% CI, 8.0–16.6) as 60.5% of patients had PD-L1 CPS ≥ 1. These outcomes were better than those of the present study, possibly due to a higher proportion of patient with PD-L1 positive status than that of our study. Overall, the addition of a PD-1 inhibitor to chemotherapy and anti-angiogenic agents in the second-line therapy showed significant superiority over chemotherapy alone or combined with anti-angiogenic agents [16].

With regard to tumor response, the ORR and DCR of a PD-1 inhibitor plus apatinib were 20.9% and 88.3%, which were better than chemotherapy alone [8, 9] or combined with anti-angiogenic agents [17]. The ORR and DCR of apatinib combined with chemotherapy were 18.52% and 92.59%, respectively [18]. By contrast, an objective response was observed in 20 (69%, 95% CI, 49–85) of 29 patients, including one patient with MMR-deficient tumor in the EPOC study [19].

The safety profile in this study was generally consistent with that of monotherapy with a PD1 inhibitor, apatinib or albumin paclitaxel, or any combination of two or three drugs for mGC (e.g., nivolumab plus paclitaxel and ramucirumab [15] or lenvatinib plus pembrolizumab [19]), with the notable exception of proteinuria, a frequent toxic effect of apatinib. The addition of a PD-1 inhibitor to apatinib did not increase the known toxic effects associated with apatinib. Common toxicities reported in this study, such as bone marrow suppression, hypertension, hand-foot reaction, and hypothyroidism, were mild to moderate and were manageable with appropriate dose modifications and supportive care. And the incidence of these AEs was similar to that reported in the EPOC1706 trial [19] and in trials involving patients with mGC [12, 15, 18]. No rare serious toxic effects occurred and no patients reported treatment-related deaths.

### Limitation

There were some limitations in our study. This was a single-armed perspective study from a single institution with limited samples resulting in some bias. As check649 and orential16 studies had published the outcome of first-line therapy with immunotherapy, therefore, a further study would be needed to investigate the efficacy of this combination for patients with prior immunotherapy history. Finally, objective response was not assessed by an independent central review, which might lead to the overestimation of anti-tumor activity results in this study.

In summary, the combination of a PD-1 inhibitor with apatinib and albumin paclitaxel showed promising efficacy and acceptable safety profile in patients with mGC who failed first-line chemotherapy. These results warranted further investigation. We look forward to a phase III clinical study to further confirm our findings.

## Data Availability

The datasets generated and/or analyzed during the current study are not publicly available due to privacy but are available from the corresponding author on reasonable request.
